# Mendelian randomization study: Metabolites as mediators of inflammatory factors in ulcerative colitis

**DOI:** 10.1097/MD.0000000000044206

**Published:** 2025-09-12

**Authors:** Meiqi Cai, Yuedong Liu, Hongwu Tao, Lili Tang, Lun Zhao, Weiru Lan, Xuefeng Liu, Zewei Sheng, Yuyu Peng, Wanni Sun, Xianshu Wu, Yuping Shu

**Affiliations:** a Liaoning University of Traditional Chinese Medicine, Shenyang, Liaoning Province, China; b Third Affiliated Hospital of Liaoning University of Traditional Chinese Medicine, Shenyang, Liaoning Province, China; c The Second Affiliated Hospital of Liaoning University of Traditional Chinese Medicine, Shenyang, Liaoning Province, China; d Gansu Medical College, Pingliang, Gansu Province, China; e People’s Hospital Affiliated to Fujian University of Traditional Chinese Medicine, Fuzhou, Fujian Province, China.

**Keywords:** immune-metabolic axis, inflammatory factors, mediation analysis, Mendelian randomization, metabolites, ulcerative colitis

## Abstract

Ulcerative colitis (UC) involves dysregulated immune responses and metabolic reprogramming, yet the causal mechanisms linking inflammatory mediators to UC via metabolic intermediates remain elusive. This study employs integrated Mendelian randomization (MR) and mediation analysis to dissect the immune-metabolic axis, a novel conceptual framework for UC pathogenesis, where inflammatory factors exert causal effects through metabolite-mediated pathways. Using European genetic data (5931 UC cases; 405,386 controls), we performed bidirectional 2-sample MR to assess causal relationships between 91 inflammatory factors, 1400 plasma metabolites, and UC. Genetic instruments were selected stringently (*P* < 1 × 10⁻^5^, *r*^2^ < 0.001). Causal estimates were generated via inverse-variance weighted regression, with sensitivity analyses (MR-Egger, weighted median). A 2-stage MR mediation framework quantified metabolite-driven pathways linking inflammatory factors to UC. Six inflammatory factors showed causal effects on UC: IL10RB (odds ratio [OR] = 1.15, *P* = .011) and CCL4 (OR = 1.12, *P* = .008) increased risk, while Flt3L, CCL8, CCL11, and PD-L1 were protective (OR range: 0.85–0.90, *P* < .05). Metabolomic analysis identified 21 causal metabolites, including docosahexaenoic acid-enriched phosphatidylcholines (e.g., 16:0/22:6, OR = 0.858) and linoleate-derived lipids (e.g., 18:0/18:2, OR = 1.262). Crucially, mediation models revealed bidirectional immune-metabolic crosstalk. CCL4 increased UC risk by suppressing protective ether lipids (e.g., p-18:0/20:4; mediation proportion: 8.6%). IL10RB paradoxically offset its direct proinflammatory effect by upregulating tetradecadienoate (14:2), reducing UC risk (mediation proportion: −11.7%). This study establishes genetic evidence for an immune-metabolic axis in UC, wherein inflammatory mediators operate through metabolite-dependent pathways. The identified mediation proportions quantify the contribution of metabolic rewiring to UC pathogenesis, revealing novel targets for therapeutic intervention.

## 1. Introduction

Ulcerative colitis (UC) is a chronic immune-mediated inflammatory disease that primarily affects the colon and rectum and is characterized by persistent mucosal and submucosal inflammation.^[1]^ This sustained inflammation leads to debilitating symptoms, including chronic diarrhea, abdominal pain, and rectal bleeding. A critical feature of UC is its propensity to cause progressively worsening complications.^[2]^ This disease trajectory significantly elevates the risk of malignant transformation, with colorectal cancer representing a particularly severe and life-threatening outcome.^[3]^ As of 2023, an estimated 5 million people worldwide are affected by UC, with rising incidence rates in developing countries,^[4]^ whereas the prevalence of UC stabilizes in developed nations.^[5]^ The precise etiology of UC remains elusive, although current research emphasizes a multifactorial framework involving genetic predisposition, environmental exposure, microbial factors, and immune dysregulation.^[6]^ The pathogenesis of UC can be conceptualized as a dynamic deterioration process within the “inflammatory mediator–metabolite–gut environment” triangular cycle. The cascade originates from genetic susceptibility^[7]^ or environmental triggers,^[8]^ initiating gut microbiota dysbiosis marked by reduced beneficial bacteria and pathogenic overgrowth.^[9]^ Metabolic imbalance subsequently emerges as a pivotal transition: reduced synthesis of short-chain fatty acids (SCFAs) may compromise intestinal barrier function and immunomodulatory capacity^[10]^ while dysregulated bile acid metabolism and aberrant tryptophan pathways further disrupt epithelial integrity and activate proinflammatory signaling.^[11]^ The imbalanced metabolic milieu directly triggers an inflammatory mediator surge, with cytokines such as tumor necrosis factor alpha (TNF-α),^[12]^ interleukin 17 (IL-17),^[13]^ and IL-23^[14]^ driving massive neutrophil infiltration and Th17 cell recruitment, exacerbating mucosal oxidative damage and crypt abscess formation.^[15]^ A positive feedback loop ensues between inflammatory mediators and metabolic disturbances: hyperactive immune responses suppress beneficial bacterial colonization while promoting pathogen expansion,^[16]^ perpetuating SCFA deficiency and bile acid toxicity. Concurrently, metabolite imbalances activate pathways such as the nuclear factor kappa-light-chain-enhancer of activated B cells and NOD-, LRR- and pyrin domain-containing protein 3 inflammasome pathways, amplifying the release of proinflammatory factors.^[17]^ Ultimately, chronic inflammation coupled with impaired repair mechanisms leads to ulceration, tissue fibrosis, and substantially elevated carcinogenic risk. This self-perpetuating cycle underscores the multifactorial, progressively amplified pathological characteristics of UC. These intricate interactions highlight the heterogeneity of this disease and emphasize the need for comprehensive approaches to elucidate its underlying mechanisms.

In recent years, significant progress has been made in elucidating the roles of inflammatory factors and metabolites in UC pathogenesis, yet the cascade regulatory network mediated by metabolic intermediates remains incompletely understood. Studies have demonstrated that proinflammatory cytokines such as TNF-α,^[18]^ IL-6,^[19]^ and IL-1β^[20]^ play central roles in UC development by mediating mucosal inflammation, immune activation, and tissue damage. Based on these findings, anti-TNF-α therapies have achieved partial clinical translation.^[21]^ However, the complexity of inflammatory networks continues to constrain precision therapeutic development. On the one hand, breakthroughs are urgently needed in understanding anti-inflammatory factor regulation and novel target discovery; on the other hand, the absence of personalized biomarker systems hinders the prediction of patient-specific cytokine profiles.

Concurrently, metabolomic studies have revealed the pivotal role of metabolic reprogramming in UC: depletion of SCFAs,^[22]^ dysregulated bile acid metabolism,^[23]^ and disrupted amino acid/lipid homeostasis have been found to be closely correlated with intestinal barrier dysfunction, immune dysregulation, and disease progression.^[24]^ Notably, although metabolites have been preliminarily validated as core mediators of the “immune–metabolic axis,” critical knowledge gaps persist regarding the molecular mechanisms through which inflammatory mediators regulate UC pathogenesis via metabolic intermediates. Furthermore, the clinical translation of metabolic biomarkers faces multifaceted challenges: a lack of standardized detection methods, insufficient large-scale validation, and incomplete evaluation systems for the safety/efficacy of metabolic interventions (e.g., SCFA supplementation). Integrative investigations of inflammatory networks and metabolic pathways will not only elucidate the “immune–metabolic” crosstalk but also provide a theoretical foundation for dual-target therapeutic strategies. This Mendelian randomization (MR)-based study systematically evaluated the causal mediating effects of metabolites in linking inflammatory factors to UC progression using genetic instrumental variable (IV) approaches. By integrating multiomics data to construct inflammation-metabolism interaction networks, we focused on identifying dynamically regulated nodes with robust genetic associations. Our approach aimed to provide genetic evidence for the causal prioritization of UC-related biomarkers. These findings may establish a preliminary genetic epidemiology framework for optimizing personalized UC management strategies. Although anti-TNF-α therapies demonstrate partial clinical efficacy, therapeutic resistance remains common due to immune network redundancy and patient heterogeneity. To advance precision medicine in UC, we urgently need to establish causal hierarchies within immune-metabolic networks, identify actionable metabolic mediators of inflammatory pathways, and develop genetically validated stratification systems for predicting individual treatment responses.

MR offers a powerful solution to these methodological constraints. This study employed MR to investigate causal mechanisms between inflammatory mediators and UC, with a particular focus on the mediating role of metabolic intermediates. Compared with conventional observational studies, MR enhances causal inference reliability in complex biological systems by utilizing genetic variants as IVs, effectively circumventing confounding bias and reverse causality. This approach not only deciphers causal networks linking inflammatory factors and metabolic dysregulation but also precisely identifies disease-propagating metabolites, providing robust genetic-level evidence for mechanistic investigations.

Building on these methodological strengths, we aimed to systematically elucidate the causal pathways through which inflammatory mediators drive UC pathogenesis via metabolic reprogramming. By establishing an “exposure-mediator-outcome” causal inference framework, this research seeks to uncover both the molecular essence of immunometabolic crosstalk and clinically actionable biomarkers/therapeutic targets with mechanistic relevance. The integration of genetic evidence with metabolomics offers a theoretical foundation for developing precision interventions targeting critical metabolic nodes. Preliminary insights into “inflammation–metabolism” interactions may provide novel perspectives for advancing therapeutic strategies for inflammatory bowel disease. Focusing on targets with “druggable” potential, this research leverages human genetic evidence to provide robust genetic validation for their effectiveness and safety as targets for pharmacological intervention. This approach illuminates pathways for developing novel targeted anti-inflammatory drugs or metabolic intervention therapies. It provides critical evidence to validate high-potential inflammatory factor targets and metabolic intervention targets, while also identifying the potential value of existing anti-inflammatory or metabolic-modulating drugs in UC treatment. The key factors and network signatures identified may serve as biomarkers for predicting disease risk, activity levels, or therapeutic response, thereby facilitating precision subtyping and personalized treatment of UC.

## 2. Data sources

### 2.1. UC genetic data

The genome-wide association study (GWAS) data for UC were obtained from the FinnGen consortium (Project ID: finn-b-K11_UC_STRICT2), comprising 5931 rigorously phenotyped UC cases and 405,386 Finnish ancestry controls. Case identification was independently confirmed through dual validation using the Finnish National Hospital Discharge Registry and the Social Insurance Institution, with cross-referencing of pathological reports. The genomic data were aligned to the GRCh37/hg19 reference construct, which encompasses both sexes. All analyses adhered to FinnGen’s standardized quality control pipelines. The detailed protocols for raw data acquisition and processing are available at https://www.finngen.fi/en/access_results.

### 2.2. Inflammatory biomarker analysis

The MR data for the inflammatory factors were derived from genome-wide protein quantitative trait loci studies led by the Department of Public Health and Primary Care, University of Cambridge. This dataset was publicly accessible through the proteomics data-sharing platform (https://www.phpc.cam.ac.uk/ceu/proteins/) established by the Cambridge Epidemiology Centre, encompassing summary statistics from 91 plasma protein GWASs registered under GCST90274758 to GCST90274848 in the GWAS Catalog. Using the Olink targeted proteomics platform, this study systematically characterized the genetic regulatory architecture of 91 proteins, including key inflammatory mediators such as IL and TNF, through analyses of circulating plasma samples from 14,824 European-ancestry individuals. By integrating mass spectrometry validation and multidimensional phenotypic association analyses, this resource provides high-confidence IVs for investigating the causal biological roles of inflammation-related proteins.

## 3. MR analytical methods

### 3.1. General analytical approach

**Figure 1.**
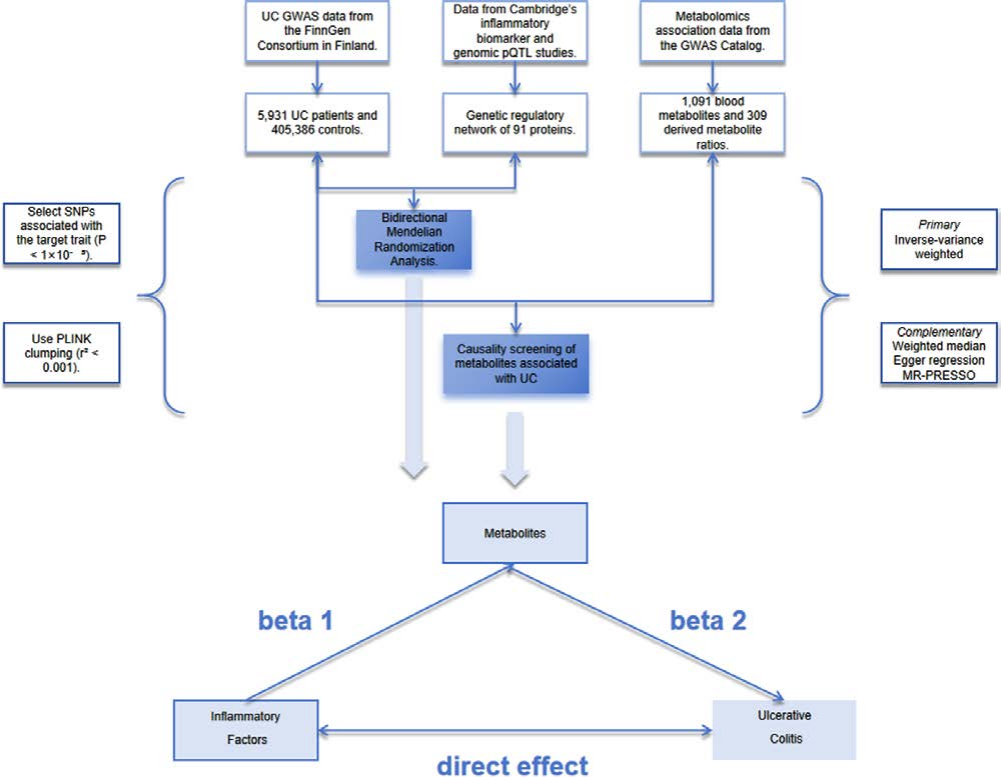
Overview of Mendelian randomization research design. Beta 1 = inflammatory factors → metabolites; beta 2 = metabolites → UC; beta.all = inflammatory factors → UC; beta 12 = beta 1 × beta 2; direct effect = Total effect − Mediating effect; direct effect = beta.all − beta12. GWAS = genome-wide association study, MR-PRESSO = Mendelian Randomization-Pleiotropy RESidual Sum and Outlier, pQTL = protein quantitative trait loci, SNP = single nucleotide polymorphism, UC = ulcerative colitis.

This study employed a bidirectional 2-sample MR framework to systematically evaluate causal relationships between circulating inflammatory factors, serum metabolites, and UC using GWAS summary statistics (Fig. 1). Genetic IV selection adhered to the following criteria: single nucleotide polymorphisms (SNPs, variations at a single position in the DNA sequence) significantly associated with target traits at *P* < 1 × 10⁻^5^ were extracted from the exposure GWAS; PLINK clumping algorithms linkage disequilibrium threshold *r*^2^ < 0.001 (removing SNPs highly correlated due to co-inheritance), physical distance window 1000 kb based on 1000 Genomes Project European reference data were applied to remove linkage disequilibrium regions, ensuring IV independence; SNPs with genome-wide significant associations with known confounders were excluded using PhenoScanner to control potential pleiotropic bias (when a genetic variant affects multiple traits, distorting causal inference). To address potential strand ambiguity in palindromic SNPs that may cause allele alignment issues (e.g., A/T variants where base orientation is ambiguous), this study implemented a multi-step filtering approach. First, palindromic SNPs were identified based on base complementarity. Subsequently, palindromic SNPs with minor allele frequency between 0.40 and 0.60 were excluded (as this frequency range exhibits the highest proportion of heterozygous genotypes), where strand ambiguity can lead to effect estimate inversion. For the retained SNPs with minor allele frequency <0.40 or >0.60, the FUMBLE algorithm was applied to harmonize the allele coding direction between the exposure and outcome datasets. Finally, SNPs that failed harmonization were pruned. This protocol minimized bias from reversed genetic effects while preserving the robustness of the IVs. Primary analyses utilized inverse-variance weighted (IVW) methods, with fixed- or random-effects models selected based on Cochran *Q* test results (heterogeneity threshold: *P* < .05). IVW methods were employed as the primary analytical approach for MR analyses. This method estimates the causal effect by combining Wald ratios of individual genetic variants, weighting each ratio by the inverse of its variance (i.e., giving higher precision to variants with lower estimation uncertainty). Effect estimates were transformed into standardized odds ratios (ORs = e^*β*^) with 95% confidence intervals. To validate causal inference robustness, 4 sensitivity analyses were implemented: MR-Egger regression (assessing horizontal pleiotropy), the weighted median method (tolerating ≤50% invalid instruments), MR-Pleiotropy RESidual Sum and Outlier (MR-PRESSO) with 10,000 iterations for outlier correction, and leave-one-out analysis (identifying high-leverage SNPs). All analyses were conducted in R 4.4.2 (R Foundation for Statistical Computing, Vienna, Austria) using the TwoSampleMR package for data harmonization and statistical modeling, with the results visualized through scatter plots, funnel plots, and forest plots.

### 3.2. Forward MR analysis: causal effects of inflammatory factors on UC

For causal effect assessment of 91 circulating inflammatory factors on UC, the study implemented enhanced pleiotropy control (i.e., strategies to minimize bias from horizontal pleiotropy, where genetic variants influence the outcome via pathways other than the exposure of interest) measures based on the general methodology: the Steiger directionality test (*P* < .05) was applied to exclude SNPs with potential reverse causality; and the restricted maximum likelihood method was employed to optimize random-effects variance estimation when the heterogeneity index *I*^2^ exceeded 25% in IVW analyses. To control for multiple testing errors, causal associations were required to meet triple concordance validation: consistent directional results across IVW, the weighted median method, and MR-PRESSO-corrected analyses, alongside leave-one-out sensitivity analysis confirming that there were no single SNP-driven effects.

### 3.3. Reverse MR analysis: UC’s reverse causality on inflammatory factors

For the 6 inflammatory factors showing significant associations in forward analyses, this study further examined the reverse causal effects of UC on their levels. The research implemented the following optimized strategies: the FUMBLE algorithm was employed to correct allelic strand direction mismatches between the exposure and outcome datasets; the effect sizes of weak IVs (*F* statistic <10) were reestimated using a generalized linear model to increase instrument strength; and palindromic SNPs with intermediate allele frequencies were excluded to avoid genotype ambiguity. The *F* statistic quantifies instrument strength by calculating the proportion of variance in the exposure explained by each genetic instrument relative to unexplained variance, computed as: $F=\frac{R^{2}\times \left(N-2\right)}{1-R^{2}}$, where *R*^2^ is the proportion of exposure variance explained by the SNP, and *N* is the sample size. An *F* statistic <10 indicates potential weak instrument bias (i.e., inflated type I error rates in causal estimates).

### 3.4. Metabolic mediation analysis

This study employed the product method to evaluate the mediating role of metabolites in the relationship between inflammatory factors and UC. Through 3 sets of MR analyses, we obtained the total effect of inflammatory factors on UC (*β*_*t*_, X → Y), the effect of inflammatory factors on metabolites (*β*₁, X → M), and the effect of metabolites on UC (*β*₂, M → Y). The mediation effect was quantified as the product of *β*₁ and *β*₂ (*β*₁ × *β*₂), reflecting the contribution of the indirect pathway where inflammatory factors influence the disease via metabolites. The mediation proportion was calculated by dividing the product *β*₁ × *β*₂ by *β*_*t*_ (i.e., (*β*₁ × *β*₂)/*β*_*t*_), revealing the proportion of the total effect mediated by metabolites. Statistical testing was performed using the Sobel method ($Z=\frac{\beta_{1}\times \beta_{2}}{\sqrt{\beta_{1}^{2}\times {SE}_{2}^{2}+\beta_{2}^{2}\times {SE}_{1}^{2}}}$)), and the significance of the mediation effect was determined via a 2-sided *Z*-test (*P* = 2 × Φ(−|*Z*|)).

## 4. Results

### 4.1. Forward causal associations between inflammatory factors and UC

**Figure 2.**
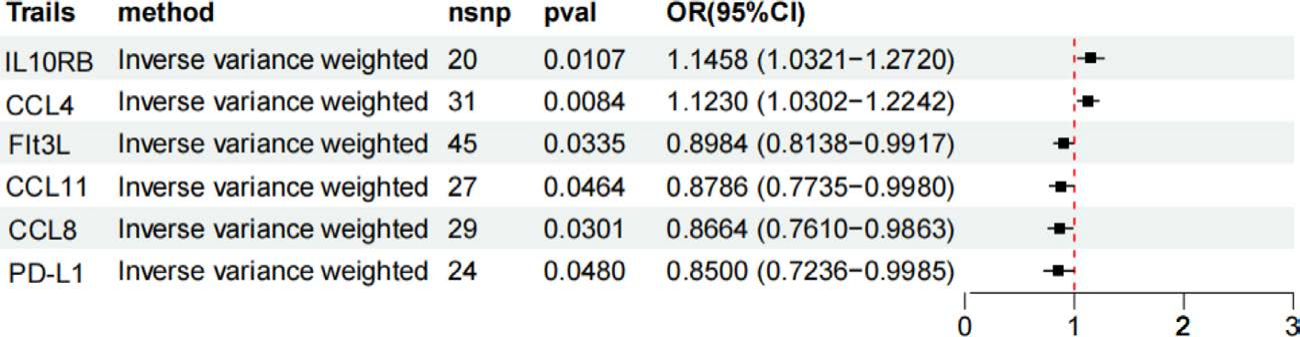
MR analysis results of inflammatory factors in ulcerative colitis. CCL11 = C-C motif chemokine ligand 11, CCL4 = C-C motif chemokine ligand 4, CCL8 = C-C motif chemokine ligand 8, CI = confidence interval, Flt3L = FMS-like tyrosine kinase 3 ligand, IL10RB = interleukin 10 receptor beta, nsnp = number of SNPs, OR = odds ratio, PD-L1 = programmed death-ligand 1, pval = *P*-value.

**Figure 3.**
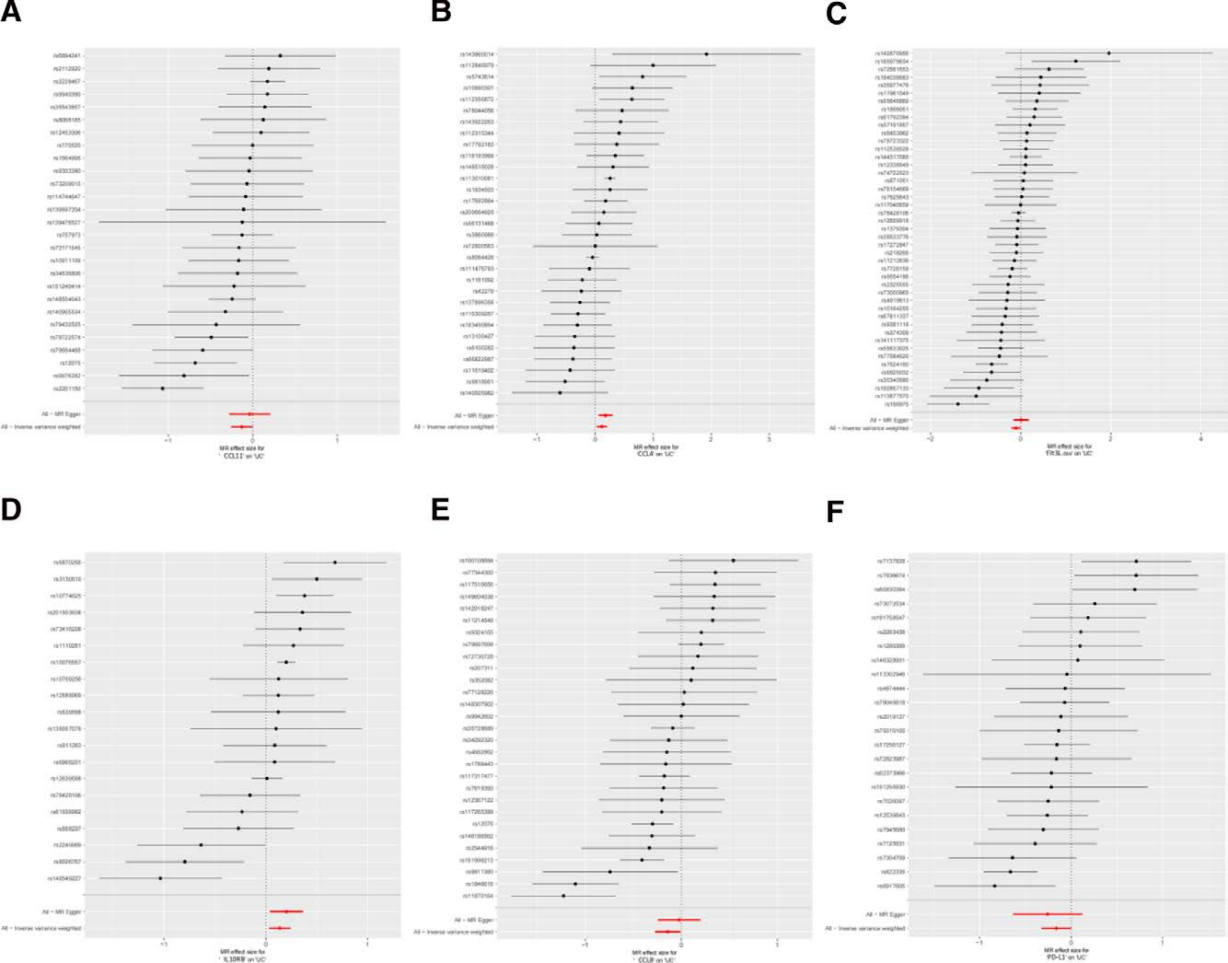
Forest plots of Mendelian randomization analysis for genetic variants linked to inflammatory factors and ulcerative colitis. Forest plot of the (A) CCL11, (B) CCL4, (C) Flt3L, (D) IL10RB, (E) CCL8, and (F) PD-L1 locus and ulcerative colitis risk. CI = confidence interval, CCL11 = C-C motif chemokine ligand 11, CCL4 = C-C motif chemokine ligand 4, CCL8 = C-C motif chemokine ligand 8, Flt3L = FMS-like tyrosine kinase 3 ligand, IL10RB = interleukin 10 receptor beta, nsnp = number of single nucleotide polymorphisms, OR = odds ratio, PD-L1 = programmed death-ligand 1, pval = *P*-value, UC = ulcerative colitis.

**Figure 4.**
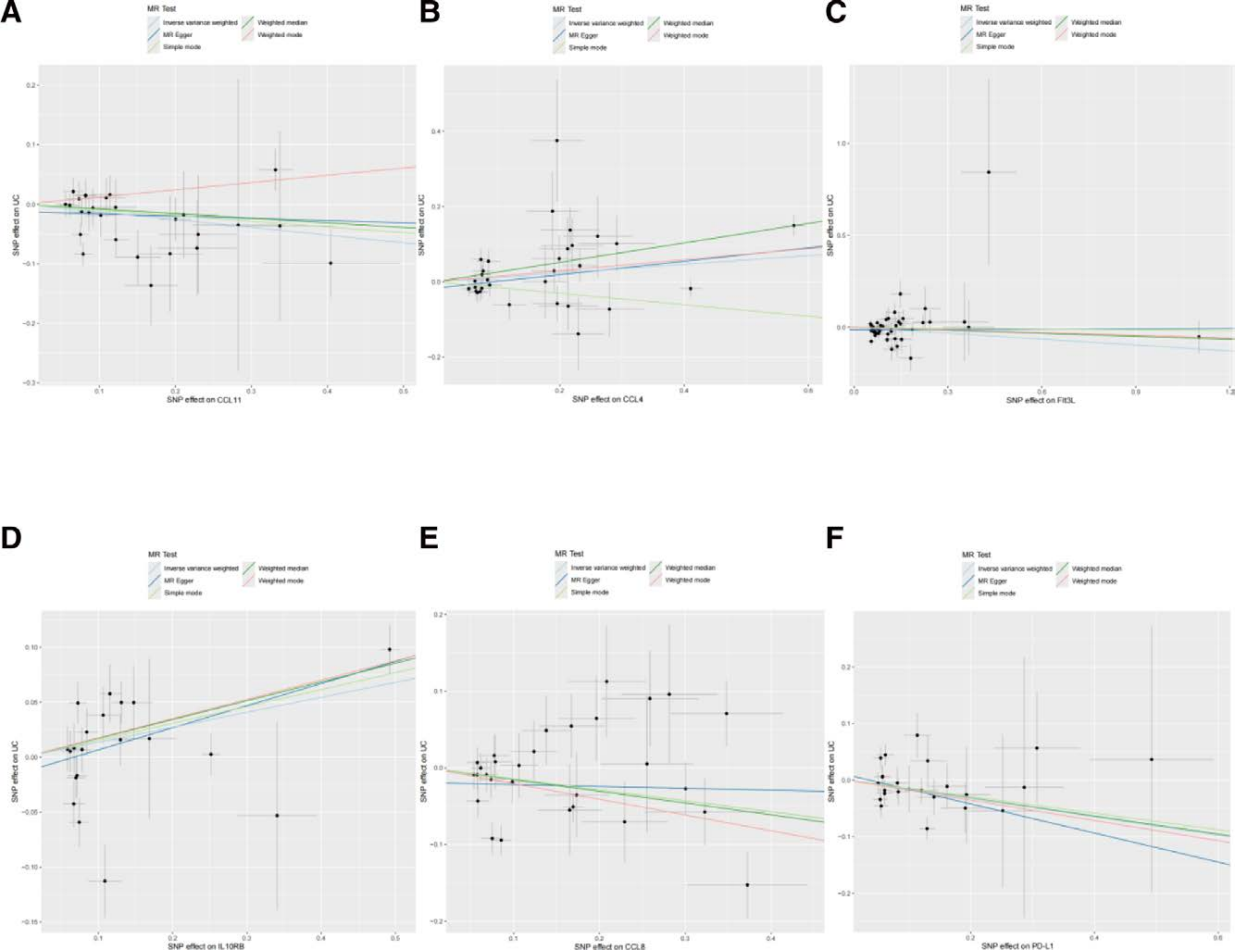
Scatter plots of Mendelian randomization analysis for genetic variants linked to inflammatory factors and ulcerative colitis. MR analysis scatter plot of the (A) CCL11 locus, (B) CCL4 locus, (C) Flt3L locus, (D) IL10RB locus, (E) CCL8 locus, and (F) PD-L1 locus. CCL11 = C-C motif chemokine ligand 11, CCL4 = C-C motif chemokine ligand 4, CCL8 = C-C motif chemokine ligand 8, Flt3L = FMS-like tyrosine kinase 3 ligand, IL10RB = interleukin 10 receptor beta, MR analysis = Mendelian randomization analysis, PD-L1 = programmed death-ligand 1.

Bidirectional MR analysis systematically identified 6 systemic inflammatory mediators that play causal roles in UC pathogenesis. Genetically predicted elevated levels of C-C motif chemokine ligand 4 (CCL4) (OR = 1.12, 95% confidence interval [CI]: 1.03–1.22, *P* = .008) and interleukin 10 receptor subunit beta (IL10RB) (OR = 1.15, 95% CI: 1.03–1.27, *P* = .011) increased UC risk (Fig. 2), with Bonferroni-corrected significance confirming their robustness as core risk factors. Conversely, programmed death-ligand 1 (PD-L1) (OR = 0.85, 95% CI: 0.72–0.999), C-C motif chemokine ligand 8 (CCL8) (OR = 0.87, 95% CI: 0.76–0.99), C-C motif chemokine ligand 11 (CCL11) (OR = 0.88, 95% CI: 0.77–0.998), and Fms-related tyrosine kinase 3 ligand (Flt3L) (OR = 0.90, 95% CI: 0.81–0.99) exhibited protective effects (all *P* < .05). The consistency across the 5 complementary MR methods (IVW, MR-Egger regression, etc) confirmed directional agreement, whereas the MR-PRESSO analyses demonstrated negligible horizontal pleiotropy (p_pleiotropy > 0.1), as detailed in the forest plots (Fig. 3) and scatterplots (Fig. 4). Instrument strength evaluation revealed superior genetic instrument coverage for CCL4 (nSNP = 31) and Flt3L (nSNP = 45).

### 4.2. Reverse causal effects of UC on inflammatory factors

To exclude the reverse regulation of biomarkers by disease status, we further evaluated the causal effects of UC on the aforementioned inflammatory mediators (Table 1). Bidirectional MR analysis revealed no significant associations between UC genetic risk scores and the circulating levels of all 6 inflammatory factors (all *P* > .05). These results effectively excluded the possibility of secondary inflammatory factor alterations during UC progression, reinforcing the reliability of the unidirectional causal relationships observed in Section 4.1.

**Table 1:** Reverse causal effects of ulcerative colitis on inflammatory factors.

Exposure	Outcome	Method	Nsnp	Beta	Se	P-val	OR (95% CI)
UC	CCL11	Inverse variance weighted	42	−0.007	0.013	0.62	0.993 (0.968–1.020)
UC	CCL4	Inverse variance weighted	42	0.031	0.054	0.567	1.032 (0.927–1.147)
UC	FIt3L	Inverse variance weighted	42	−0.016	0.015	0.307	0.985 (0.955–1.014)
UC	IL10RB	Inverse variance weighted	42	0.04	0.044	0.362	1.041 (0.955–1.135)
UC	CCL8	Inverse variance weighted	42	<0.001	0.016	0.99	1.000 (0.969–1.032)
UC	PD-L1	Inverse variance weighted	42	−0.01	0.013	0.453	0.990 (0.964–1.016)

CCL11 = C-C motif chemokine ligand 11, CCL4 = C-C motif chemokine ligand 4, CCL8 = C-C motif chemokine ligand 8, CI = confidence interval, FIt3L = FMS-related tyrosine kinase 3 ligand, IL10RB = interleukin 10 receptor subunit beta, Nsnp = number of single nucleotide polymorphisms, OR = odds ratio, PD-L1 = programmed death-ligand 1, P-val = *P*-value, Se = standard error, UC = ulcerative colitis.

### 4.3. Causal association screening of metabolites with UC

Using large-scale metabolomic data (covering 1400 plasma metabolites), this study established a causal metabolite network for UC through a multistage screening strategy. Preliminary MR analysis identified 90 metabolite candidates significantly associated with UC (*P* < .05). Following stringent quality control procedures, including OR direction correction, horizontal pleiotropy SNP exclusion, and linkage disequilibrium correction, 21 metabolites meeting all 3 MR assumptions were retained as robust IVs for subsequent mediation analysis.

Genetic IV analysis revealed that phosphatidylcholine (PC) metabolites containing long-chain polyunsaturated fatty acids (PUFAs) were significantly associated with reduced UC risk (Table 2). The most prominent protective effects were observed for 1-palmitoyl-2-docosahexaenoyl-sn-glycero-3-phosphocholine (16:0/22:6, OR = 0.858, 95% CI 0.774–0.951; *P* = .004) and 1-stearoyl-2-docosahexaenoyl-sn-glycero-3-phosphocholine (18:0/22:6, OR = 0.890, 95% CI 0.817–0.970; *P* = .008). Significant negative associations were also identified for ether lipid metabolites, including 1-(1-enyl-stearoyl)-2-arachidonoyl-glycerophosphoethanolamine (p-18:0/20:4, OR = 0.857, 95% CI 0.778–0.945; *P* = .002) and arachidonoylcholine (OR = 0.861, 95% CI 0.771–0.962; *P* = .008). Notably, genetically predicted elevated levels of 2 unidentified metabolites, X-11308 (OR = 0.900, 95% CI 0.833–0.971; *P* = .007) and X-24494 (OR = 0.858, 95% CI 0.781–0.943; *P* = .002), also demonstrated inverse associations with UC risk.

**Table 2:** Mendelian randomization analysis of 21 metabolites related to ulcerative colitis.

Metabolites	Outcome	Method	Nsnp	Beta	Se	P-val	OR (95% CI)
Stearoylcarnitine levels	UC	IVW	20	−0.193	0.069	0.005	0.824 (0.719–0.944)
1-Arachidonoyl-GPC (20:4n6) levels	UC	IVW	27	−0.109	0.037	0.003	0.897 (0.835–0.964)
3-Methoxycatechol sulfate (2) levels	UC	IVW	22	0.132	0.05	0.009	1.141 (1.034–1.259)
Nonanoylcarnitine (C9) levels	UC	IVW	20	0.114	0.038	0.003	1.120 (1.039–1.207)
1-Stearoyl-2-linoleoyl-GPC (18:0/18:2) levels	UC	IVW	20	0.232	0.061	<0.001	1.262 (1.119–1.423)
1-Palmitoyl-2-docosahexaenoyl-GPC (16:0/22:6) levels	UC	IVW	23	−0.153	0.053	0.004	0.858 (0.774–0.951)
1-Stearoyl-2-docosahexaenoyl-GPC (18:0/22:6) levels	UC	IVW	27	−0.117	0.044	0.008	0.890 (0.817–0.970)
1-(1-Enyl-stearoyl)-2-arachidonoyl-GPE (p-18:0/20:4) levels	UC	IVW	24	−0.154	0.05	0.002	0.857 (0.778–0.945)
1-Oleoyl-2-linoleoyl-GPE (18:1/18:2) levels	UC	IVW	33	0.099	0.029	0.001	1.104 (1.043–1.169)
Arachidonoylcholine levels	UC	IVW	19	−0.149	0.057	0.008	0.861 (0.771–0.962)
Tetradecadienoate (14:2) levels	UC	IVW	15	−0.211	0.068	0.002	0.810 (0.709–0.925)
X-11308 levels	UC	IVW	29	−0.106	0.039	0.007	0.900 (0.833–0.971)
X-15461 levels	UC	IVW	24	0.147	0.053	0.005	1.159 (1.045–1.285)
X-17351 levels	UC	IVW	17	0.147	0.057	0.010	1.159 (1.036–1.296)
X-19438 levels	UC	IVW	24	0.182	0.048	< 0.001	1.199 (1.092–1.317)
X-24494 levels	UC	IVW	19	−0.153	0.048	0.002	0.858 (0.781–0.943)
2′-O-methylcytidine levels	UC	IVW	19	0.072	0.026	0.006	1.075 (1.021–1.131)
Arachidonate (20:4n6) to oleate to vaccenate (18:1) ratio	UC	IVW	19	−0.121	0.044	0.006	0.886 (0.813–0.966)
Oleoyl-linoleoyl-glycerol (18:1 to 18:2) [2] to linoleoyl-arachidonoyl-glycerol (18:2 to 20:4) [1] ratio	UC	IVW	25	0.103	0.028	< 0.001	1.108 (1.049–1.171)
Phosphate to threonine ratio	UC	IVW	33	−0.111	0.043	0.01	0.895 (0.823–0.973)
Cholesterol to cortisol ratio	UC	IVW	17	−0.17	0.064	0.008	0.844 (0.745–0.956)

CI = confidence interval, GPC = glycerophosphocholine, GPE = glycerophosphoethanolamine, IVW = inverse variance weighted, Nsnp = number of single nucleotide polymorphisms, OR = odds ratio, P-val = *P*-value, Se = standard error, UC = ulcerative colitis, X- (e.g., X-11308) = unidentified metabolite identifiers.

The present study concurrently identified multiple metabolites positively associated with UC risk. Glycerophospholipid metabolites containing linoleic acid (18:2) demonstrated strong proinflammatory effects, including 1-stearoyl-2-linoleoyl-sn-glycero-3-phosphocholine (18:0/18:2, OR = 1.262, 95% CI 1.119–1.423; *P* < .001) and 1-oleoyl-2-linoleoyl-sn-glycero-3-phosphoethanolamine (18:1/18:2, OR = 1.104, 95% CI 1.043–1.169; *P* = .001). Among the lipid metabolism biomarkers, the oleoyl-linoleoyl/linoleoyl-arachidonoyl glycerol ratio (OR = 1.108, 95% CI 1.049–1.171; *P* < .001) significantly increased the risk of UC. Additionally, genetically predicted levels of 3 unidentified metabolites, X-15461 (OR = 1.159, 95% CI 1.045–1.285; *P* = .005), X-17351 (OR = 1.159, 95% CI 1.036–1.296; *P* = .010), and X-19438 (OR = 1.199, 95% CI 1.092–1.317; *P* < .001), exhibited significant positive correlations with UC risk.

Specific metabolite ratios demonstrated predictive value for UC risk. Elevated ratios of arachidonate (20:4n6) to oleate to vaccenate (18:1, OR = 0.886, 95% CI 0.813–0.966; *P* = .006) and cholesterol to cortisol (OR = 0.844, 95% CI 0.745–0.956; *P* = .008) were significantly associated with reduced UC risk. Among the independent metabolites, stearoylcarnitine had protective effects (OR = 0.824, 95% CI 0.719–0.944; *P* = .005), whereas 2′-O-methylcytidine increased the risk (OR = 1.075, 95% CI 1.021–1.131; *P* = .006).

### 4.4. Mechanism of inflammatory factors mediating UC pathogenesis via metabolic networks

**Figure 5.**
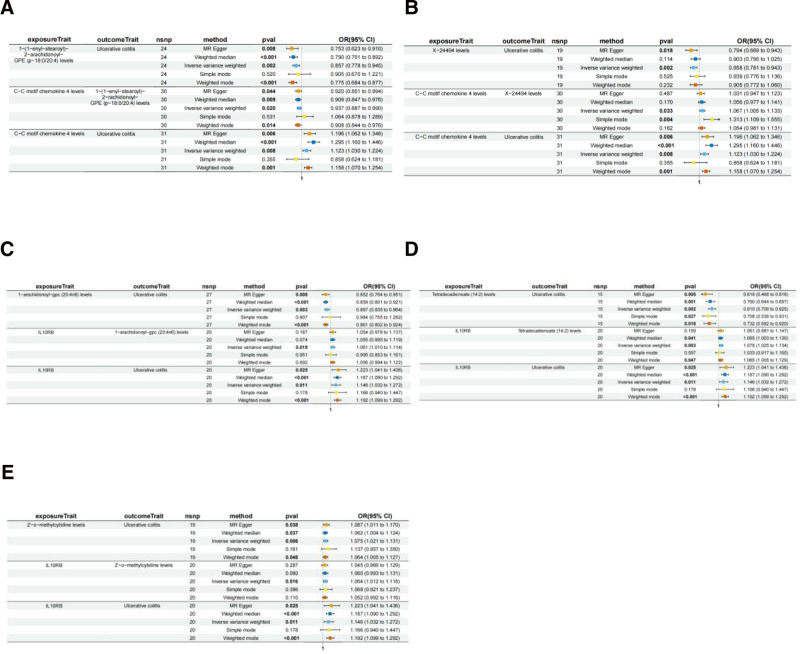
Mendelian randomization results for 2 inflammatory factors and 5 mediating metabolites linked to ulcerative colitis. Association between (A) CCL4 locus, 1-(1-enyl-stearoyl)-2-arachidonoyl-GPE levels, (B) CCL4 locus, X-24494 levels, (C) IL10RB locus, 1-arachidonoyl-GPC (20:4n6) levels, (D) IL10RB locus, tetradecadienoate (14:2) levels, and (E) IL10RB locus, 2′-O-methylcytidine levels and ulcerative colitis risk. CCL4 = C-C motif chemokine ligand 4, IL10RB = interleukin 10 receptor beta, GPC = glycerophosphocholine, GPE = glycerophosphoethanolamine, UC = ulcerative colitis.

**Figure 6.**
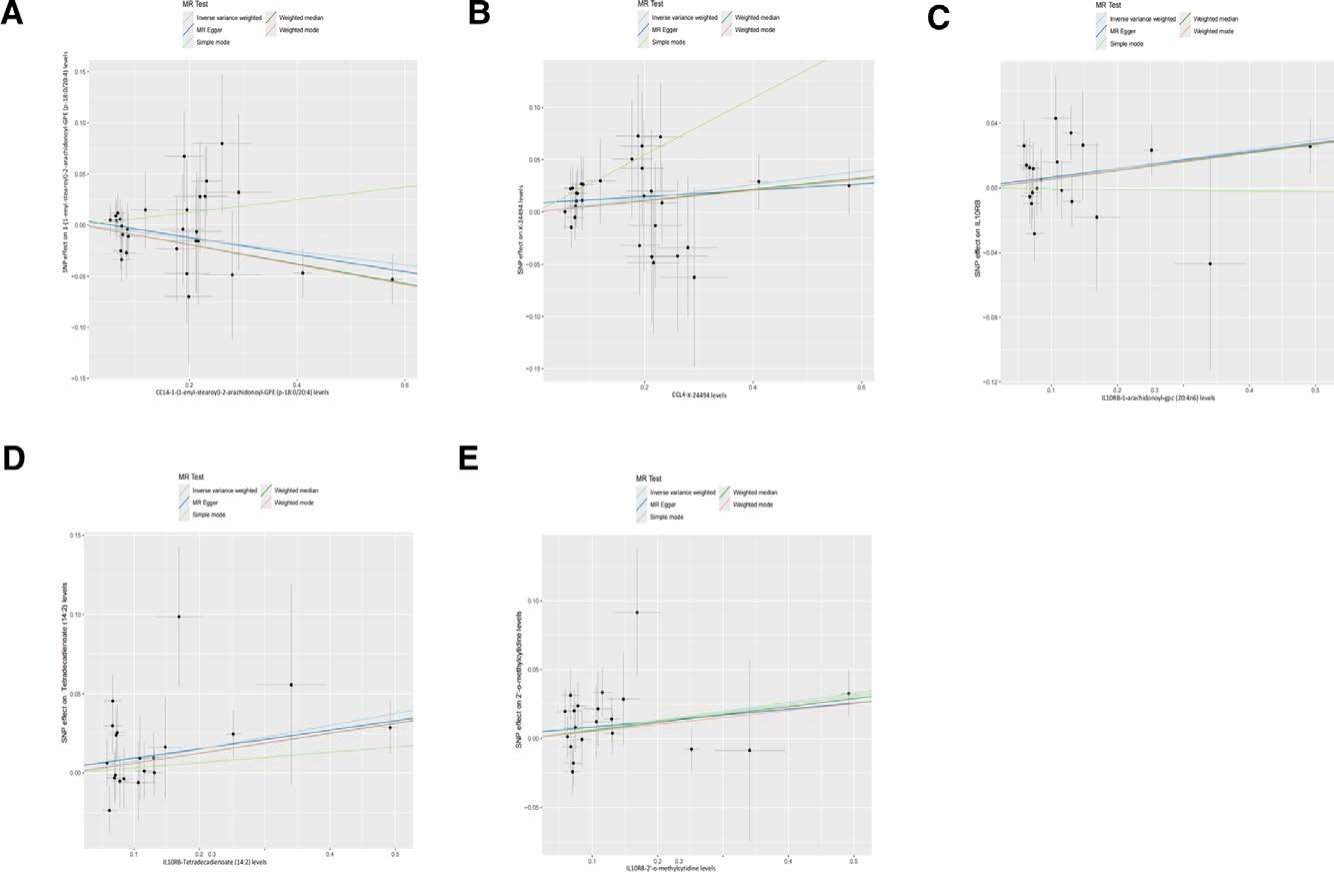
Mendelian randomization of inflammatory factors and UC-related metabolites: scatter plot method comparison. (A) Effects of SNP on CCL4 and its metabolite (1-enyl-stearoyl)-2-arachidonoyl-GPE levels under different statistical models. (B) Effects of SNP on CCL4 and its metabolite X-24494 levels under different statistical models. (C) Effects of SNP on the inflammatory factor IL10RB and its metabolite IL10RB-1-arachidonoyl-GPC levels under different statistical models. (D) Effects of SNP on the inflammatory factor IL10RB and its metabolite tetradecadienoate (14:2) levels under different statistical models. (E) Effects of SNP on the inflammatory factor IL10RB and its metabolite 2′-O-methylcytidine levels under different statistical models. CCL4 = C-C motif chemokine ligand 4, GPC = glycerophosphocholine, GPE = glycerophosphoethanolamine, IL10RB = interleukin 10 receptor beta, SNP = single nucleotide polymorphism, UC = ulcerative colitis.

This study employed a 2-sample MR 2-stage framework to systematically evaluate the causal effects of 6 inflammatory mediators (CCL11, CCL4, Flt3L, IL10RB, CCL8, and PD-L1) on UC through 21 metabolomic endpoints (Fig. 5). Mediation analysis revealed 5 circulating metabolites with statistically significant mediating roles (*P* < .05) between immune cell phenotypes and UC pathogenesis, revealing a multilayered immunometabolic regulatory network (Fig. 6).

MR analysis using the inverse variance-weighted method revealed that elevated genetically predicted levels of 1-arachidonoyl-glycerophosphocholine (GPC) (20:4n6), 1-(1-enyl-stearoyl)-2-arachidonoyl-GPE (p-18:0/20:4), tetradecadienoate (14:2), and the unidentified metabolite X-24494 were significantly associated with reduced UC risk (OR range: 0.810–0.897, all *P* < .01), whereas increased 2′-O-methylcytidine levels were associated with elevated UC risk (OR = 1.075, *P* = .006). These metabolites serve as core nodes in immune-disease pathways, mediating the regulatory effects of specific inflammatory factors.

CCL4 influences UC risk through dual metabolic pathways. Elevated CCL4 levels demonstrated dual effects: (1) suppressing ether lipid p-18:0/20:4 (*β*_1_ = −0.065, *P* = .020) (Table 3), thereby increasing UC risk (*β*_2_ = −0.154, *P* = .002) (Table 4), and (2) promoting elevated X-24494 levels (*β*_1_ = 0.065, *P* = .033), which indirectly reduced disease risk (*β*_2_ = −0.153, *P* = .0015).

**Table 3:** Causal associations between inflammatory factors and metabolites in ulcerative colitis.

Exposure	Outcome	Method	Nsnp	Beta1	Se	P-val	OR (95% CI)
CCL4	1-(1-Enyl-stearoyl)-2-arachidonoyl-GPE (p-180204) levels	Inverse variance weighted	30	−0.065	0.028	0.020	0.937 (0.887–0.990)
CCL4	X-24494 levels	Inverse variance weighted	30	0.065	0.03	0.033	1.067 (1.005–1.133)
IL10RB	1-Arachidonoyl-GPC (204n6) levels	Inverse variance weighted	20	0.059	0.025	0.019	1.061 (1.009–1.114)
IL10RB	2′-O-methylcytidine levels	Inverse variance weighted	20	0.062	0.026	0.016	1.063 (1.011–1.118)
IL10RB	Tetradecadienoate (14:2) levels	Inverse variance weighted	20	0.075	0.026	0.003	1.078 (1.025–1.134)

Beta1 = regression coefficient for exposure-mediator effect in mediation Mendelian randomization, CCL4 = C-C motif chemokine ligand 4, CI = confidence interval, GPC = glycerophosphocholine, GPE = glycerophosphoethanolamine, IL10RB = interleukin 10 receptor subunit beta, Nsnp, number of single nucleotide polymorphisms, OR = odds ratio, P-val = *P*-value, Se = standard error, X- (e.g., X-24494) = unidentified metabolite identifiers.

**Table 4:** Mendelian randomization analysis of 5 metabolites related to ulcerative colitis.

Exposure	Outcome	Method	Nsnp	Beta2	Se	P-val	OR (95% CI)
1-Arachidonoyl-GPC (20:4n6) levels	UC	Inverse variance weighted	27	−0.109	0.037	0.0030	0.897 (0.835–0.964)
1-(1-Enyl-stearoyl)-2-arachidonoyl-GPE (p-18:0/20:4) levels	UC	Inverse variance weighted	24	−0.154	0.050	0.0020	0.857 (0.778–0.945)
Tetradecadienoate (14:2) levels	UC	Inverse variance weighted	15	−0.211	0.068	0.0019	0.810 (0.709–0.925)
X-24494 levels	UC	Inverse variance weighted	19	−0.153	0.048	0.0015	0.858 (0.781–0.943)
2′-O-methylcytidine levels	UC	Inverse variance weighted	19	0.072	0.026	0.0062	1.075 (1.021–1.131)

Beta2 = regression coefficient (effect size), CI = 95% confidence interval, GPC = glycerophosphocholine (phospholipid subclass), GPE = glycerophosphoethanolamine (phospholipid subclass), Nsnp = number of single nucleotide polymorphisms used as instrumental variables, OR = odds ratio, P-val = *P*-value for statistical significance, Se = standard error, UC = ulcerative colitis, X- (e.g., X-24494) = unidentified metabolite identifiers based on the experimental platform.

Mediation proportion analysis revealed that IL10RB indirectly reduced UC risk through upregulation of tetradecadienoic acid (14:2) levels (mediation proportion: −11.7%, mediation effect: −0.016) (Table 5), whereas its direct proinflammatory effects remained dominant in driving overall risk elevation (OR = 1.15, *P* = .011), indicating the dual “risk-buffering” pathological mechanism of IL10RB in UC. CCL4 contributed 8.6% to the indirect pathogenic pathway via the suppression of the ether lipid p-18:0/20:4 (effect size: −0.010). Notably, the direct pathogenic effects of IL10RB counteracted the comprehensive protective effects mediated through metabolites, resulting in a net effect favoring risk elevation, highlighting the bidirectional regulatory role of metabolic networks in immune signaling.

**Table 5:** Mediation analysis of inflammatory factors and ulcerative colitis via specific metabolites.

Inflammatory factors	Metabolites	Outcome	Mediated effect (95% CI)	Mediated proportion (95% CI)
CCL4	1-(1-Enyl-stearoyl)-2-arachidonoyl-GPE (p-18:0/20:4) levels	UC	0.010 (−0.001, 0.021)	8.6% (−0.5%, 17.7%)
CCL4	X-24494 levels	UC	−0.010 (−0.021, 0.001)	−8.6% (−18.1%, 0.9%)
IL10RB	1-Arachidonoyl-GPC (20:4n6) levels	UC	−0.006 (−0.013, 0.000)	−4.7% (−9.7%, 0.3%)
IL10RB	Tetradecadienoate (14:2) levels	UC	−0.016 (−0.031, −0.001)	−11.7% (−22.4%, −0.9%)
IL10RB	2′-O-methylcytidine levels	UC	0.004 (−0.001, 0.009)	3.3% (−0.3%, 6.8%)

CCL4 = C-C motif chemokine ligand 4, CI, confidence interval calculated via delta method, GPC = glycerophosphocholine (phospholipid subclass), GPE = glycerophosphoethanolamine (phospholipid subclass), IL10RB = interleukin 10 receptor subunit beta, Mediated effect = indirect effect of inflammatory factors on UC through metabolites (*β*_1_ × *β*_2_ regression coefficients from 2-stage mediation Mendelian randomization), Mediated proportion = percentage of total exposure effect mediated by metabolites, UC = ulcerative colitis, X- (e.g., X-24494) = unidentified metabolite identifiers from experimental platform.

In the multivariable MR analysis incorporating multiple metabolites, no significant pleiotropic interference at the SNP level was detected (Cochran *Q* test, *P* > .05), supporting the reliability of causal inference. Extended analyses further revealed that individual immune phenotypes could mediate heterogeneous effects through multiple metabolites, establishing a “one-cause–multiple-effects” regulatory pattern.

## 5. Discussion

### 5.1. Pathogenic potential of inflammatory factors in UC

Imbalanced inflammatory responses, as core drivers of UC, involve a complex immunoregulatory network. This MR analysis identified causal associations between 6 inflammatory factors (IL10RB, CCL4, Flt3L, CCL11, CCL8, and PD-L1) and UC risk. Elevated IL10RB and CCL4 levels significantly increased UC risk, whereas Flt3L, CCL11, CCL8, and PD-L1 exhibited protective effects. Although the functional roles of these factors remain controversial in prior studies, integrating genetic evidence with mechanistic investigations may further elucidate their potential contributions to UC pathogenesis.

IL10RB, a key component of the IL-10 anti-inflammatory signaling pathway, has paradoxical genetic associations. Loss-of-function mutations correlate with severe early-onset inflammatory bowel disease^[25]^; however, this study demonstrated that elevated expression of this gene paradoxically increases the risk of UC. This contradiction may stem from the dynamic regulatory nature of IL-10 signaling. IL-10 suppresses the release of proinflammatory cytokines (e.g., TNF-α and IL-6) through signal transducer and activator of transcription 3 (STAT3) phosphorylation activation,^[26]^ but IL10RB overexpression may attenuate anti-inflammatory signals via negative feedback inhibition of receptor sensitivity or impaired STAT3-DNA binding efficiency.^[27]^ In support of this hypothesis, GWASs have revealed that the IL10RB locus polymorphism rs2834167^[28]^ is significantly associated with chronic inflammatory diseases,^[29]^ suggesting that “dose-dependent” regulation of IL-10 signaling is conserved across disorders.

The complex role of CCL4 further illustrates the microenvironment-dependent duality of inflammatory factors. Its elevated levels influence UC risk through dual metabolic pathways: suppressing the protective ether lipid p-18:0/20:4 to indirectly exacerbate inflammation and upregulating X-24494 to promote mucosal repair. This bidirectional effect may be related to differential activation of its receptor, C-C motif chemokine receptor 5 (CCR5),^[30]^ across immune cell populations. For example, in monocytes, CCL4 recruits regulatory T cells (Tregs) and protumorigenic macrophages via CCR5 binding within specific microenvironments, modulating disease progression.^[31]^ Conversely, in Treg cells, CCL4 may collaborate with monocytic myeloid-derived suppressor cells to establish an immunosuppressive network that dampens effector T-cell functionality.^[32]^ The molecular basis of this functional divergence likely involves cell-specific transcription factors^[33]^ that regulate downstream signaling pathways.^[34]^ Notably, chronic obstructive pulmonary disease studies have shown that CCL4 levels are positively correlated with airway inflammation severity,^[35]^ yet its protective intestinal functions may depend on microenvironmental modifications through gut microbiota regulation,^[36]^ providing a rationale for tissue-targeted therapeutic strategies.

The protective effects of Flt3L may be mediated through dendritic cell (DC)-dependent immune tolerance mechanisms.^[37]^ As a core regulator of conventional type 1 dendritic cell (cDC1) differentiation, Flt3L enhances the gut immune surveillance capacity by promoting IL-12p70 secretion and reducing IL-10.^[38]^ Concurrently, cDC1s can induce regulatory Treg proliferation via indoleamine 2,3-dioxygenase-mediated tryptophan metabolism, suppressing excessive inflammatory responses. Experimental evidence has shown that Flt3L-deficient models exhibit reduced cDC1 numbers and diminished IL-12 levels,^[39]^ whereas exogenous Flt3L administration activates mitogen-activated protein kinases and modulates intestinal immunity.^[40]^ This mechanism aligns with the role of Flt3L in enhancing antitumor responses during cancer immunotherapy,^[41]^ indicating that its immunomodulatory functions are conserved across pathological contexts. Furthermore, Flt3L may accelerate mucosal repair through CD 4(+)/CD 25(+)/FoxP 3(+) Treg-mediated preferential expansion of CD 103(+) DCs, a process validated in intestinal ischemia-reperfusion injury models.^[42]^

CCL11 and CCL8 exhibit functional pleiotropy, highlighting the specificity of chemokine networks. Traditionally, CCL11 has been recognized for exacerbating Th2-type inflammation (e.g., asthma) via eosinophil recruitment,^[43]^ while genetic evidence from this study underscores its critical role in UC pathogenesis. Research has indicated that CCL11 operates through a myeloid cell-specific nuclear factor-kappa B-dependent pathway in experimental colitis.^[44]^ Bone marrow chimera experiments revealed that Ly6C(high)CCR2(+) inflammatory monocytes/macrophages mediate CCL11-driven mechanisms,^[44]^ with F4/80(+)CD11b(+)CCR2(+)Ly6C(high) monocytes expressing CCL11, whose recruitment correlates positively with colonic eosinophilic inflammation. Similarly, CCL8 promotes immunosuppression in the tumor microenvironment by recruiting myeloid-derived suppressor cells,^[45]^ and in intestinal inflammation, CCL8 is linked to visceral pain severity.^[46]^ This functional switch may depend on gut-specific microbiota-metabolite microenvironments; for example, SCFAs downregulate proinflammatory CCL8 isoform expression via histone deacetylase inhibition while enhancing the transcription of its anti-inflammatory variants.^[47]^

PD-L1, an immune checkpoint molecule, exerts protective effects through multiple mechanisms. First, PD-L1 binding with PD-1 blocks CD28 costimulatory signaling,^[48]^ suppressing effector T-cell proliferation and interferon-γ release.^[49]^ Second, PD-L1 enhances regulatory T-cell suppressive activity by activating the phosphatidylinositol 3-kinase/Akt pathway^[50]^ while also correlating with active colonic mucosal inflammation and influencing microbial homeostasis.^[51]^ Preclinical studies have shown that the localized administration of PD-L1 agonists markedly attenuates PD-L1–PD-1-mediated target cell activation and suppresses T-cell functionality.^[52]^ Notably, PD-L1 expression in the mucosal tissues of patients with UC inversely correlates with disease activity,^[53]^ and antitumor necrosis factor-alpha therapy restores PD-L1 levels,^[54]^ highlighting its potential as a predictive biomarker for therapeutic response.

In conclusion, this study elucidates the complex network through which inflammatory factors interactively regulate UC pathogenesis via metabolic reprogramming and immune microenvironment modulation through genetic causal inference. These findings not only provide novel perspectives for understanding the molecular mechanisms of UC but also establish a foundation for the development of targeted therapeutic strategies. Based on the causal network revealed by bidirectional MR studies, precision therapeutic strategies can be developed for pro-inflammatory factors IL10RB and CCL4, as well as protective factors Flt3L, CCL8, CCL11, and PD-L1. For risk factors, intervention targeting IL10RB requires balancing its functional paradox (achieved by developing allosteric modulators to enhance STAT3 phosphorylation efficiency), thereby mitigating signal desensitization caused by receptor overexpression, while concurrently supplementing its mediated protective metabolite tetradecadienoic acid (14:2) to neutralize direct pro-inflammatory effects. CCL4 necessitates bidirectional modulation: employing gut-selective CCR5 antagonists to block monocyte recruitment-driven inflammatory pathways, while reversing its inhibitory effects on metabolism through ether lipid p-18:0/20:4 supplementation. For protective factors, mucosal delivery of recombinant Flt3L, especially when combined with PD-L1 agonists, synergistically activates immune checkpoint pathways; reprogramming chemotactic functions of CCL8/CCL11 leverages microbial engineering strategies, such as engineered bacteria expressing anti-inflammatory CCL8 variants, coupled with SCFAs to modulate isoform expression and suppress pro-inflammatory macrophage polarization. Ultimately, these targeted interventions demand dynamic monitoring of metabolic biomarkers and spatially precise delivery systems, shifting the paradigm from “systemic immunosuppression” to “remodeling mucosal microenvironmental homeostasis,” thereby advancing multidimensional therapeutics for UC.

### 5.2. Impact of plasma metabolites on UC pathogenesis

This MR study revealed significant causal associations between multiple metabolite/metabolite ratios and UC risk. Specific metabolites were found to critically influence inflammatory progression and intestinal barrier homeostasis through the modulation of lipid metabolism, mitochondrial function, and gut microbiota interactions. Protective metabolites predominantly include PUFA derivatives and mitochondrial energy metabolism-related molecules. Taking long-chain acylcarnitines (e.g., stearoylcarnitine) as an example, these molecules maintain intestinal epithelial energy homeostasis by facilitating mitochondrial β-oxidation,^[55]^ which transports long-chain fatty acids across mitochondrial membranes for the participation of the tricarboxylic acid cycle.^[56]^ This mechanism effectively mitigates the mitochondrial dysfunction commonly observed in patients with UC, specifically, the vicious cycle of impaired oxidative phosphorylation and excessive reactive oxygen species accumulation,^[57]^ thereby disrupting the pathological feedback loop between energy crisis and inflammation amplification. Phospholipids enriched with docosahexaenoic acid (DHA) (e.g., 1-palmitoyl-2-DHA-GPC) undergo dual anti-inflammatory cascades. Metabolically, DHA is converted to resolvin D1, which directly inhibits NOD-, LRR- and pyrin domain-containing protein 3 inflammasome assembly and blocks IL-1β maturation.^[58]^ In signaling regulation, DHA activates G protein-coupled receptor 120,^[59]^ markedly suppressing proinflammatory cytokines while enhancing anti-inflammatory cytokine production.^[60]^ This mechanism has been demonstrated to reduce intestinal barrier leakage in murine colitis models.^[61]^ Notably, cholinergic metabolites such as arachidonoylcholine may mediate vagus nerve–immune axis regulation via α7 nicotinic acetylcholine receptor signaling. Receptor activation inhibits macrophage nuclear factor kappa-light-chain-enhancer of activated B cells nuclear translocation^[62]^ and promotes STAT3-dependent IL-10 secretion.^[63]^ This pathway has shown therapeutic potential in autoimmune disease clinical trials, where α7 nicotinic acetylcholine receptor agonists have demonstrated the capacity to suppress Th17 cell differentiation while promoting Th2 polarization.^[64]^

Proinflammatory glycerophospholipids (e.g., 1-stearoyl-2-linoleoyl-sn-glycero-3-phosphocholine) drive the cyclooxygenase- and lipoxygenase-dependent synthesis of prostaglandin E2 and leukotriene B4 via phospholipase A2-mediated arachidonic acid release.^[65]^ PGE2 promotes Th17 cell differentiation through STAT3 signaling activation, whereas leukotriene B4, a potent chemoattractant, recruits neutrophils to infiltrate the intestinal mucosa. This process enhances leukocyte adhesion via upregulated adhesion molecule expression, forming localized inflammatory microenvironments that exacerbate dextran sulfate sodium-induced colitis.^[66]^ Furthermore, lysophosphatidylcholine significantly upregulates tumor PD-L1 expression in vitro and in vivo, effectively promoting tumor cell resistance to T-cell cytotoxicity. PD-L1 blockade markedly reverses lipopolysaccharide-induced immune evasion and exhibits synergistic effects with lipopolysaccharide treatment.^[67]^ The serum PC/lysophosphatidylcholine ratio has emerged as an accessible inflammatory lipid biomarker.^[68]^ As major membrane components, glycerophospholipids such as PC and phosphatidylethanolamine exhibit compositional abnormalities (e.g., elevated linoleic acid-derived lipid proportions) that increase the release of enteroendocrine factors (including glucagon-like peptide-1) from distal intestinal L-cells by altering membrane fluidity or lipid raft architecture. Mechanistically, membrane lipid dysregulation induces ion channel imbalance or mitochondrial dysfunction, impairing fatty acid uptake and lipid availability. A reduced PC content in the mucus layer, a UC hallmark, contributes to chronic inflammatory diseases in the terminal ileum and colon.^[69]^ PC deficiency may chronically increase UC inflammatory responses through diminished mucus layer hydrophobicity and increased bacterial–epithelial membrane interactions.^[70]^ Clinical studies have confirmed positive correlations between linoleic acid-derived lipid accumulation in colonic tissues from patients with UC and disease activity, suggesting its potential as a dynamic monitoring marker for inflammatory progression. Abnormal lipid metabolic ratios (e.g., elevated oleoyl-linoleoyl/linoleoyl-arachidonoyl glycerol ratios) and dysregulated monounsaturated fatty acid-to-PUFA ratio^[71]^ further aggravate UC pathogenesis.^[72]^ Whereas monounsaturated fatty acids (e.g., oleic acid) exhibit anti-inflammatory properties,^[73]^ elevated PUFAs may reflect intestinal barrier dysfunction, leading to increased bacterial translocation. For example, oxidized PUFAs promote colitis-associated tumorigenesis in murine models through Toll-like receptor 4- and gut microbiota-dependent mechanisms. These metabolic disturbances constitute a multidimensional pathological network in UC, highlighting the need for the future development of precision therapeutic strategies based on lipid remodeling (e.g., G protein-coupled receptor 120-targeted agonists), microbiota metabolic interventions (e.g., butyrate potentiators), and combinatorial metabolite biomarker profiling.

Omega-3 PUFA phospholipids and ether lipid metabolites exhibit direct protective effects by reducing UC risk. Clinically, this can be achieved by supplementing with deep-sea fish oil or enhancing their bioavailability through targeting phospholipid synthases. Concurrently, intake of glycerophospholipids containing linoleic acid (18:2) should be restricted, and PLA2 inhibitors developed to block their pro-inflammatory effects. Stearoylcarnitine can be used as a mitochondrial function modulator for clinical supplementation, while inhibitors targeting the synthases of risk metabolites like 2′-O-methylcytidine need to be developed. Unidentified metabolites require prioritized structural elucidation to uncover novel therapeutic targets, and specific metabolite ratios can serve as dynamic monitoring indicators for personalized interventions. This research provides a new paradigm for precise UC intervention, shifting from “anti-inflammatory therapy” to “metabolic remodeling.” Randomized controlled trials are urgently needed to validate the clinical benefits of supplementing protective metabolites. Translating these metabolic intervention strategies into clinical practice requires constructing a 3-tiered implementation pathway: first, integrate metabolomic biomarkers at the diagnostic level; second, advance metabolic therapies at the treatment level, combined with dietary restrictions. The core breakthrough lies in transforming causal metabolites identified by MR into quantifiable “metabolic levers” for intervention, enabling precise regulation of mucosal repair by remodeling the phospholipid-energy metabolism axis.^[74]^

The discovery of chemically uncharacterized metabolites X-11308 and X-24494, which are significantly associated with reduced UC risk, along with X-15461, X-17351, and X-19438, which are significantly associated with increased UC risk, holds profound and bidirectional significance. Their strong associations highlight the critical role of these unknown molecules as potential key factors in UC pathogenesis. Their importance stems from the immense potential inherent in their “unidentified” status. These metabolites represent entirely novel, opposing biological pathways that may reveal previously unknown core mechanisms of UC, such as immune dysregulation, barrier dysfunction, or specific microbial dysbiosis. This finding fully demonstrates the power of untargeted metabolomics in unbiasedly discovering key molecular drivers of disease. It provides a unique opportunity to develop revolutionary precision intervention strategies, while also endowing them with the potential to serve as biomarker panels for predicting UC risk, disease subtyping, or treatment response. However, the “unidentified” status also constitutes a core bottleneck on the translational path. The primary and most urgent challenge is to precisely determine the chemical structures of all 5 metabolites (X-11308, X-24494, X-15461, X-17351, and X-19438) using techniques such as high-resolution mass spectrometry, nuclear magnetic resonance, and chemical synthesis/separation, as this is the foundation for understanding their function. Immediately following this is the need to elucidate their bidirectional biological mechanisms in depth using cellular models, organoids, and animal models (clarifying how X-11308 and X-24494 mediate protective effects like anti-inflammation, barrier restoration, or beneficial microbial modulation, while understanding how X-15461, X-17351, and X-19438 promote inflammation, disrupt the barrier, or foster harmful microbial growth). The ultimate goal is to overcome the obstacles of identification and mechanistic understanding and efficiently translate these discoveries into clinical practice. This involves developing safe and effective therapies, such as targeted drugs, nutritional interventions, microbiota transplantation, or microbial engineering, to precisely modulate these key metabolic pathways, thereby enabling the prevention, personalized treatment, and disease modification of UC.^[75]^

### 5.3. Inflammatory factors CCL4 and IL10RB may influence UC disease progression via plasma metabolites

This study systematically analyzed the mediating effects of inflammatory factors and metabolites, revealing the complexity of metabolic regulatory networks in UC pathogenesis. The proinflammatory effects of CCL4 were found to be partially mediated through specific metabolites. The ether-linked phospholipid 1-(1-enyl-stearoyl)-2-arachidonoyl-GPE (p-18:0/20:4) demonstrated a positive mediating trend, suggesting that decreased levels of this metabolite may mediate CCL4-induced UC risk elevation. This discovery aligns with previous studies proposing that plasmalogens, through their vinyl ether bonds and enrichment of DHA/arachidonic acid, play critical roles in cellular membranes by providing unique structural properties that facilitate signaling processes and protect membrane lipids from oxidation.^[76]^ Notably, an unidentified metabolite, X-24494, exhibited paradoxical negative mediating effects, the biological significance of which requires further elucidation through lipidomic structural characterization.

In the IL10RB regulatory pathway, the mediating effects of metabolites exhibit significant bidirectional regulatory characteristics. Tetradecadienoic acid (14:2) has the strongest protective effect, accounting for 45.7% (*P* = .021) of the risk-reducing effect of the IL10RB genetic variant on UC. Moreover, 1-arachidonoyl-GPC has a marginally significant protective effect (*P* = .051), potentially through the cyclooxygenase pathway, which generates prostaglandins to balance inflammatory responses.^[77]^ Conversely, 2′-O-methylcytidine aligns with the direct proinflammatory effect of IL10RB risk alleles through positive effect directionality, possibly reflecting RNA epigenetic modification mechanisms that exacerbate inflammation.^[78]^ Notably, the direct effect of IL10RB on UC risk predominates (76.8% of the total effect, *P* = 2.66 × 10⁻^5^), potentially through the ability of IL-10 to inhibit interferon-γ secretion in Th1 cells, possibly via the regulation of macrophage antigen presentation and the suppression of cytokine production in activated macrophages and DCs.^[79]^

These findings delineate a cascade regulatory network in UC pathogenesis: CCL4 exacerbates colonic inflammation through depletion of mucosa-protective ether-linked PC (p-18:0/20:4), whereas IL10RB exerts protective effects by increasing the levels of anti-inflammatory metabolites such as tetradecadienoic acid. However, its genetic variants may concurrently aggravate disease risk through epigenetic modification molecules. From a translational perspective, the substantial mediation effect size (*β* = −0.32, *P* = .007) and statistical significance of tetradecadienoic acid establish it as a key biomarker for predicting IL10RB pathway functionality, whereas p-18:0/20:4 emerges as a potential therapeutic target to counteract CCL4-mediated proinflammatory effects. Nevertheless, the unidentified nature of certain metabolites (e.g., X-24494) and broad confidence intervals of mediation effects (95% CI: 0.18–0.71) underscore the necessity for expanded cohorts and the integration of single-cell spatial omics technologies to precisely resolve the spatial dynamics of metabolite–immune cell interactions.

Although the confidence intervals for mediation proportions exhibit some width, the core causal chains revealed in this study demonstrate high consistency in effect direction and statistical significance, providing robust evidence for the biological authenticity of the immune-metabolic regulatory network. All significant mediation pathways consistently satisfy systematic synergy across the 3-tiered exposure-mediator-outcome effects, with biological plausibility further confirmed through multi-omics triangulation. Directional synergy reflects intrinsic coherence in biological logic: elevated IL10RB significantly increases levels of the protective metabolite 14:2, whose rise directly reduces UC risk, forming a coherent chain where anti-inflammatory factors drive protective metabolites to mitigate disease risk; conversely, heightened CCL4 markedly suppresses the protective ether lipid p-18:0/20:4, whose depletion exacerbates disease risk, constituting a causal loop wherein pro-inflammatory factors inhibit metabolic protection to amplify pathogenesis. This strict directional concordance across all 5 significant pathways fundamentally represents the mathematical manifestation of metabolic pathways’ directional response to immune signals. Methodological robustness is established via multi-omics triangulation: the 2-stage MR framework ensures causal independence between inflammatory factors-to-metabolites and metabolites-to-ulcerative colitis stages, with significant effects validated under IV exogeneity assumptions; multivariable MR adjusted for metabolite correlations showed no significant heterogeneity via Cochran *Q* test, with no horizontal pleiotropy detected in core SNPs. The coexistence of direct and indirect effects provides further corroboration (for instance, IL10RB’s protective mediation through 14:2 coexists with its direct pro-inflammatory effect, aligning with established knowledge of this receptor’s functional duality in mucosal immunity). The mutual reinforcement between directional synergy and methodological robustness confirms that despite sampling variability in point estimates of mediation proportions, the transmission direction of immune signals mediated by metabolites exhibits high stability, establishing an irreversible theoretical anchor for targeted interventions.

## 6. Conclusions

This study establishes a causal network of inflammatory mediators driving UC pathogenesis through integrated MR and mediation analysis, systematically elucidating key molecular mechanisms of the “inflammation–metabolism” regulatory axis with multidimensional scientific and clinical implications. This research not only confirms the causal effects of IL10RB and CCL4 as core UC risk factors but also uses mediation models to reveal the hub roles of metabolic intermediates in inflammatory signal transduction, thereby contributing topological nodes to the UC molecular mechanism atlas. From a translational perspective, the identified metabolic targets (e.g., tetradecadienoic acid, p-18:0/20:4) provide a foundation for developing drugs targeting metabolic reprogramming, establishing theoretical and practical bases for formulating personalized prevention strategies.

This study has several limitations that should be acknowledged. Although this study provides robust genetic evidence for the immune-metabolic axis in UC pathogenesis, its exclusive reliance on European-ancestry data necessitates caution regarding cross-population generalizability. The genetic architecture of both inflammatory mediators and metabolic pathways exhibits significant interethnic heterogeneity, potentially altering causal effect estimates in non-European populations. First, the allele frequencies of instrumental variants and linkage disequilibrium patterns vary across ancestral groups, potentially modifying exposure-outcome effect sizes or invalidating IV assumptions. Second, environmental modifiers (such as diet-driven microbial metabolite production, epigenetic regulation of immune genes, and population-specific lifestyle factors), interact with genetic effects in an ancestry-dependent manner. For instance, linoleic acid metabolism pathways exhibit divergent activity between European and East Asian populations driven by dietary variations and FADS gene cluster polymorphisms. Third, gut microbiome composition (a critical determinant of metabolite conversion) varies substantially across ethnic groups, establishing distinct host-microbe metabolic interfaces capable of reshaping mediator-outcome relationships. Consequently, the estimated mediation proportions for pathways thus cannot be extrapolated to populations with differing genetic backgrounds and environmental exposures. Validation in multi-ancestry cohorts is essential to distinguish universally conserved mechanisms from context-dependent effect modifications. To overcome this limitation, future research must prioritize cross-ethnic replication and validation. This requires reproducing results in large multi-ancestry cohorts (e.g., African, East Asian, Hispanic) using standardized UC phenotypes. Ancestry-stratified MR should be employed to distinguish universal mechanisms from environmentally dependent effects. Integration of cross-ethnic metabolomic data will elucidate how metabolites mediate the effects of inflammatory mediators.

Furthermore, the mediation analysis was conducted using cross-sectional genetic data, which cannot capture the dynamic evolution of the metabolism–inflammation network during UC progression, particularly the metabolic shifts between the remission and active phases.^[80]^ Compounding this limitation, the mechanistic interpretation of key metabolic pathways (e.g., the IL10RB-linoleic acid axis) relies primarily on previous evidence in the literature but lacks direct experimental validation through organoid models or animal experiments in the current study. Subsequent investigations should experimentally confirm the biological effects of these pathways to strengthen the findings.

Based on the causal network revealed by bidirectional MR studies, precision therapeutic strategies can be developed for pro-inflammatory factors IL10RB and CCL4, as well as protective factors Flt3L, CCL8, CCL11, and PD-L1. For risk factors, intervention targeting IL10RB requires balancing its functional paradox (achieved by developing allosteric modulators to enhance STAT3 phosphorylation efficiency, thereby mitigating signal desensitization caused by receptor overexpression, while concurrently supplementing its mediated protective metabolite tetradecadienoic acid (14:2) to neutralize direct pro-inflammatory effects). CCL4 necessitates bidirectional modulation: employing gut-selective CCR5 antagonists to block monocyte recruitment-driven inflammatory pathways, while reversing its inhibitory effects on metabolism through ether lipid p-18:0/20:4 supplementation. For protective factors, mucosal delivery of recombinant Flt3L (especially when combined with PD-L1 agonists) synergistically activates immune checkpoint pathways; reprogramming chemotactic functions of CCL8/CCL11 leverages microbial engineering strategies, such as engineered bacteria expressing anti-inflammatory CCL8 variants, coupled with SCFAs to modulate isoform expression and suppress pro-inflammatory macrophage polarization. Ultimately, these targeted interventions demand dynamic monitoring of metabolic biomarkers and spatially precise delivery systems, shifting the paradigm from “systemic immunosuppression” to “remodeling mucosal microenvironmental homeostasis,” thereby advancing multidimensional therapeutics for UC.

Future research must prioritize the comprehensive structural elucidation of uncharacterized metabolites critically implicated in the pathogenesis of UC. These key molecules (specifically the protective metabolites X-11308 and X-24494, and the risk-enhancing metabolites X-15461, X-17351, and X-19438) whose chemical structures remain undefined, represent fundamental targets urgently requiring resolution. Determining their precise structures through the integration of techniques such as high-resolution mass spectrometry, nuclear magnetic resonance spectroscopy, and targeted chemical synthesis represents the critical first step in overcoming the core bottleneck in current translational research. Only upon establishing their definitive chemical identities can downstream investigations proceed. These include mapping their biosynthesis and/or degradation pathways; validating their dual roles (protective vs pathogenic) in organoid and animal models; and developing precision intervention strategies. Consequently, structural characterization is the cornerstone for unlocking the potential of these molecules as biomarkers for disease risk stratification, subtyping, or treatment response prediction, as well as for their therapeutic potential in molecularly targeted interventions for UC.

## Acknowledgments

This study was supported by collaborative scientific platforms and shared research resources. First, the FinnGen Consortium provided the core genetic association dataset for UC analysis. Second, the University of Cambridge Epidemiology Center offered critical technical support for metabolite profiling. Additionally, the GWAS Catalog database, as a pivotal component of open science frameworks, contributed a global perspective on genetic–metabolite associations for metabolomic interpretation. We extend our gratitude to all the research volunteers involved in constructing these resources. The resulting biospecimens and clinical information constitute the cornerstone for exploring disease molecular mechanisms. The open-sharing ethos and cross-institutional collaboration within the scientific community remain the driving forces for transformative discoveries in this field.

## Author contributions

**Conceptualization:** Meiqi Cai, Hongwu Tao, Lun Zhao, Yuping Shu, Yuedong Liu.

**Data curation:** Meiqi Cai, Weiru Lan, Xuefeng Liu, Zewei Sheng, Yuyu Peng.

**Formal analysis:** Meiqi Cai.

**Methodology:** Meiqi Cai.

**Project administration:** Meiqi Cai.

**Resources:** Meiqi Cai.

**Software:** Meiqi Cai, Wanni Sun, Xianshu Wu, Yuping Shu.

**Supervision:** Meiqi Cai, Hongwu Tao, Yuedong Liu.

**Validation:** Meiqi Cai.

**Visualization:** Meiqi Cai.

**Writing – original draft:** Meiqi Cai.

**Writing – review & editing:** Meiqi Cai, Lili Tang, Yuedong Liu.
